# Supplementary material for: Sleep fragmentation exacerbates myocardial ischemia‒reperfusion injury by promoting copper overload in cardiomyocytes
Source: Nat Commun. 2024 May 7;15:3834. doi: 10.1038/s41467-024-48227-y (PMC11076509; doi:10.1038/s41467-024-48227-y)
Supplement: Supplementary file 1 — Supplemental Information [file 41467_2024_48227_MOESM1_ESM.pdf]

Sleep fragmentation exacerbates myocardial ischemia–reperfusion injury by promoting copper overload in cardiomyocytes

Na Chen[1], Lizhe Guo[1], Lu Wang[1], Sisi Dai[1], Xiaocheng Zhu[1], E Wang[*1,2]

Supplemental Material

Supplementary Table 1 List of antibodies

Antibodies	CAT. No	Company	Dilution
Rabbit Anti-Caspase-3 p12	ab179517	Abcam	1:1000
Mouse Anti-PDC-E2	sc-271534	SANTA	1:200-1:400
Mouse anti-Hsp70	ab2787	Abcam	1:1000
Rabbit anti-Lipoic Acid	ab58724	Abcam	1:2000
Rabbit anti-ADX	ab108257	Abcam	1:4000
Rabbit anti-GPX4	ab125066	Abcam	1:4000
Mouse Anti-ATP7A	sc-376467	SANTA	1:400-1:100
Rabbit anti-BAX	ab32503	Abcam	1:4000
Rabbit anti-BCL-2	ab196495	Abcam	1:1000
Rabbit anti-VPS35	ab157220	Abcam	1:10000
Rabbit anti-GAPDH	YN5585	IMMUNOWAY	1:5000
Rabbit anti-ATOX1	ab154179	Abcam	1:5000
Rabbit anti-Flag	K200001M	Solarbio	1:1000
Rabbit anti-β-Tubulin	AP0064	Bioworld	1:5000

Goat Anti-Mouse IgG 488	ab150113	Abcam	1:1000
Goat Anti-Rabbit IgG 488	ab150077	Abcam	1:1000
Goat Anti-Mouse IgG 594	ab150116	Abcam	1:1000
Goat Anti-Rabbit IgG(H+L)	SA00001-2	Proteintech	1:10000
Anti-Mouse IgG(H+L)	SA00001-1	Proteintech	1:10000

Supplementary Table 2 List of primer sequence

Gene	Primer sequence(Forward)	Primer sequence(Reverse）
Gapdh	TGTGTCCGTCGTGGATCTGA	CCTGCTTCACCACCTTCTTGA
Bcl-2	ATGCCTTTGTGGAACTATATGGC	GGTATGCACCCAGAGTGATGC
Bax	TGAAGACAGGGGCCTTTTTG	AATTCGCCGGAGACACTCG
Casp8	ACCAAATGAAGAACAAACCTCG	CTTCATTTTTCGGAGTTGGGTT
Fdx-1	TCCACTTCAAGAACCGAGATGG	CAAGTAGAGCAAGCCAACGTTC
Ripk1	CTCACCACTGACAGCCAGACAATG	AAGGGCACACACCATGAGAGTAAAC
Ripk3	TCCACACTCCGAGCCGTAGAC	CCAGAGGAACCGCATAACTTGACAG
Mlkl	ACAGGCTACACCATTCGGAAACAC	TCTGCTTTAGTGCTCTTTGCTGTCC
Dlat	ACCTCAGGCATGTATTCTGGCAATC	CTCCAACTGCTCCATCCACAACTC
Atp7a	GGGATGACCTGTGCTTCTTGTGTAG	TTACTTCTGCCTTGCCAGCCATTAG
Sdhb	GCTACTGGTGGAACGGAGACAAG	TGGCAGCGGTAGACAGAGAAGG
Aco2	GTGGGTGGTGATTGGAGATGAGAAC	CCTGGCGAAGCTCTTGGTGATG
Slc31a	TTCGCTACAATTCCATGCCTGTCC	GCTGATGACTACCTGGATGATGTGC
Lias	GGTGGCTCTGTCTGGATTAGATGTG	TGGACCTCCTTGGCATGTCTCAG

Gpx4	CCCGATATGCTGAGTGTGGTTTAC	TTTCTTGATTACTTCCTGGCTCCTG
Th	AGTTTGACCCTGACCTGGACCTG	ATTGGCTCACCCTGCTTGTATTGG
Ntf3	CACCACGGAGGAAACGCTATGC	AATGTCAATGGCTGAGGACTTGTCG
Vps35	GCTACGTTCTGATGATCCTGACCAG	AAGGTGGCAGTGTGAAGCGAATC
Ngf	AGTGCCGAGCCTCCAATCCTG	GTGTGAGTCGTGGTGCAGTATGAG
Sema3a	TGGGACGGGACTTCGCTATCTTC	TCAGGGTTGTCACTCTCTGGGATG
Snx27	CGAGCAGGCGAGAAGGAATTG	AGTCGTCACTGGGATCTAGGTTATC
Vps18	CTGCGTCCATGTCTATAAGGTGTTG	TTGCGAAGTTCCTCATCCTCCTC
Snx12	GCGGACAAACCTACCCATCTTC	GCAGTGGTGGTACTACGATCTTAC
AP-1c	CCAACGACCTGCCTTCTGATGC	GTTCTGGACACACGGGCTAATGG
Ptbp1	CCAGGCTCCAAGAACTTCCAGAAC	TTTGACCACACCACCGTTGCTG
LOX-1	ACACAGAGGAGAGTGGCTGAAGG	ATGTCTGCCGCATAGGTGTCATAAC
Atox1	TCTCCGTGGACATGACCTGTGAG	AGAGTCGATGCAGACCTTCTTGTTG
Cog1	CCGGAGCAGCACTTTCAACT	CCCTCGCCTTTCTTGTGCTT
Grx1	TCTGGGAAGGTGGTCGTGTT	GCACTGGTGTTGTTAGTGGCT
Akt2	CCGCCCAGTCCATCACAATCAC	TCGGATGCTGGCTGAGTAGGAG
Commd1	GCAGAGCCGCTGGGACAAC	GTATCTGAGTTGAGTGCCGTGACTG

Supplemental Figures and Figure Legends:

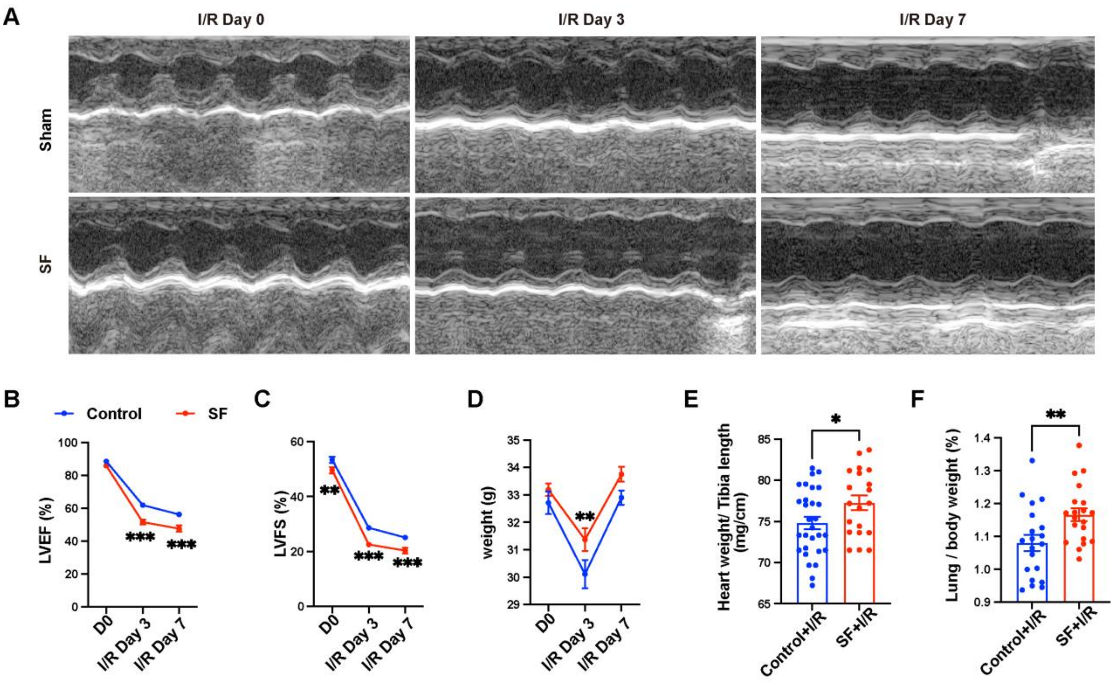

Fig. S1 Chronic SF exacerbated MI/RI in mice. (A) Representative M-mode echocardiographic changes in mice with SF and control mice. **(B-D)** Trend chart of changes in LVEF, LVFS and body weight between mice with SF and control mice on day 3 and day 7 after MI/RI (n=10 mice per group). (**E)** Heart weight/tibia length ratio (n=30 mice per group). (**F)** Lung weight/body weight ratio (n=20 mice per group). Data are presented as the mean ± s.e.m. The significance of differences was evaluated using an unpaired t test compared with the control group at the same time point. *P<0.05, **P<0.01, ***P<0.001.

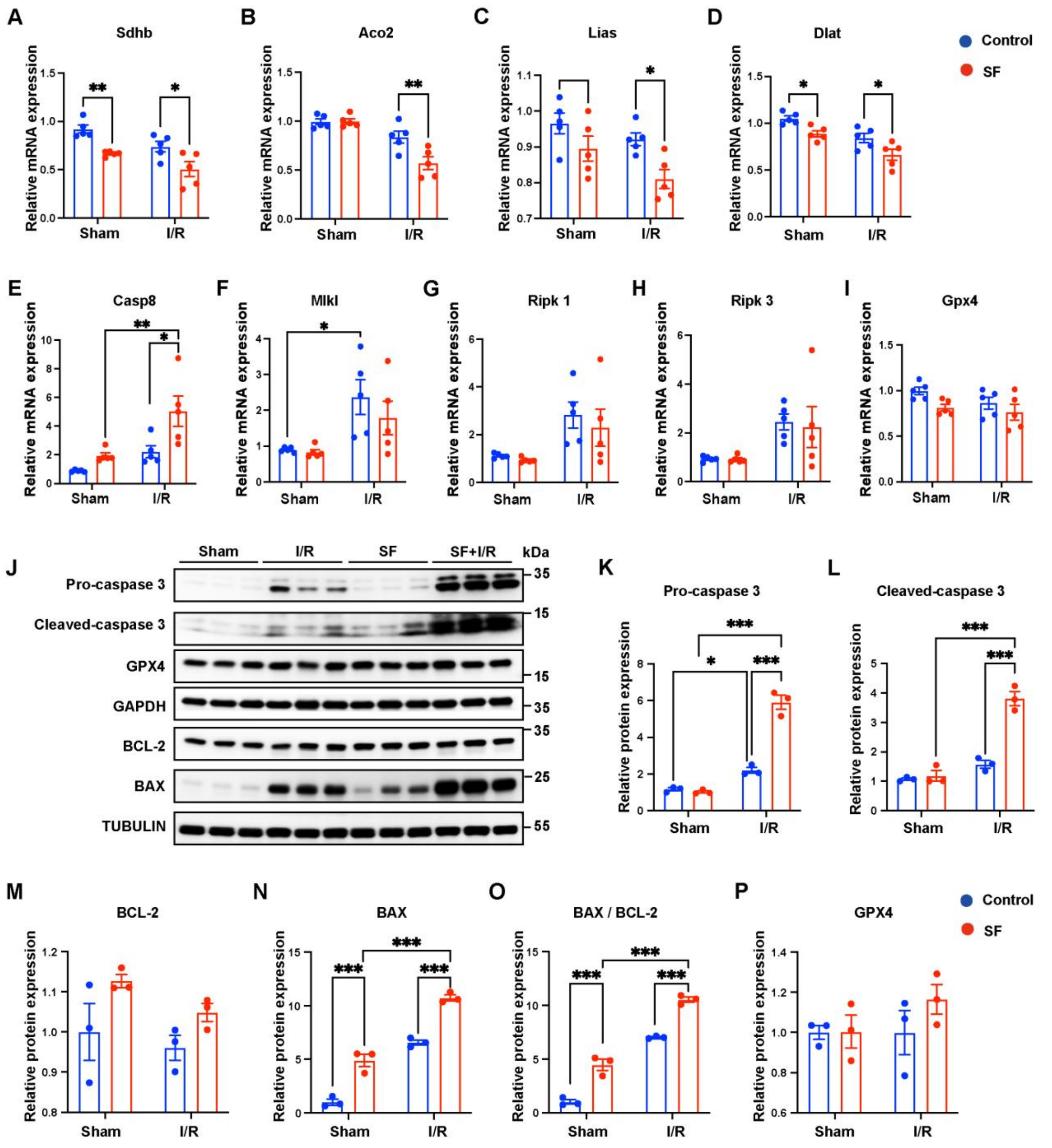

Fig. S2 Chronic SF exacerbated cuproptosis and apoptosis in mice. (A-I) RT–qPCR analyses of the mRNA levels of Sdhb, Aco2, Lias, Dlat, Casp8, Mlkl, Ripk1, Ripk3 and Gpx4 in the myocardium (n=5 mice per group). (**J**) Validation of the levels of pro-caspase 3, cleaved caspase 3, BCL-2, BAX and GPX4 by Western blotting. **(K-P)** Statistical analysis of pro-caspase 3, cleaved caspase 3, BCL-2, BAX and GPX4 expression. Data on pro-caspase 3, cleaved-caspase 3 and GPX4 were normalized to GAPDH, while data on

BCL-2 and BAX were normalized to tubulin (n=3 mice per group). Data are presented as the mean ± s.e.m. The significance of differences was evaluated using one-way ANOVA. *P<0.05, **P<0.01, ***P<0.001.

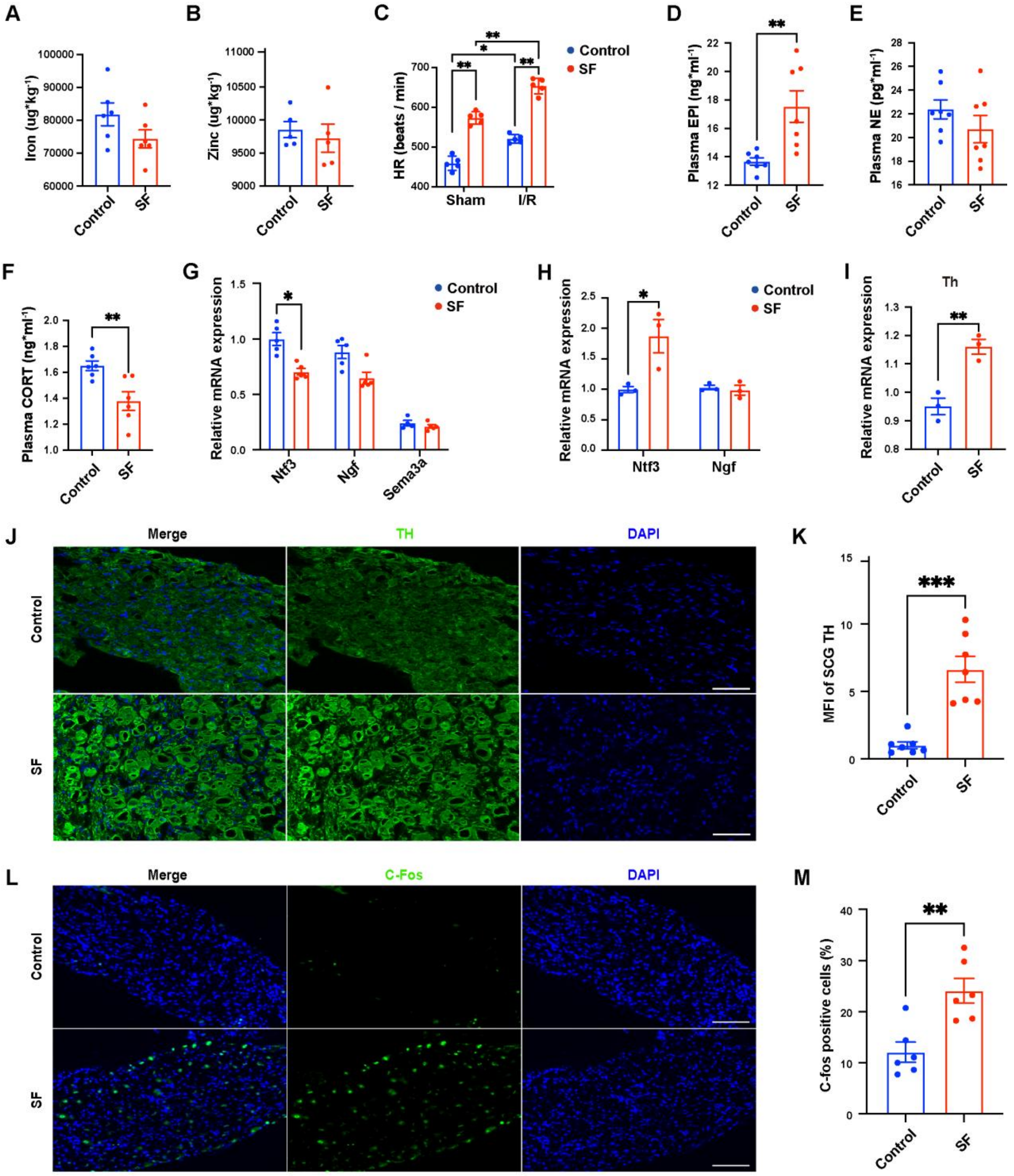

Fig. S3 Fragmented sleep led to sympathetic hyperactivity. (A-B) ICP–MS was used to detect essential trace elements. In contrast to copper

ions, iron ions and zinc ions did not show differences in abundance between the two groups (n=5 mice per group). **(C)** Cardiac electrophysiology telemetry found that SF mice show faster resting heart rate (HR) in SF mice of both sham and MI/RI group (n=5 mice per group). (**D-F)** ELISA was performed to detect epinephrine (EPI), norepinephrine (NE) and corticosterone (CORT) in the plasma of mice with SF and control mice (n=8 mice per group). (**G)** RT–qPCR analyses of the mRNA levels of Ntf, Ngf and Sema3a in the myocardium (n=5 mice per group). **(H-I)** RT–qPCR analyses of the mRNA levels of Ntf, Ngf and Th in the SCG (n=3 mice per group). **(J-K)** Representative wide-field and confocal immunofluorescence images of TH in the SCG by confocal microscopy. TH was quantified by calculating the mean fluorescence intensity (n=7 mice per group). Scale bar=50 μm. **(L-M)** Averaged data on C-FOS positive cells were calculated as the ratio of C-Fos-positive nuclei to DAPI-stained nuclei (n=6 mice per group). Scale bar=50 μm. Data are presented as the mean ± s.e.m. The significance of differences was evaluated using an unpaired t test. *P<0.05, **P<0.01, ***P<0.001.

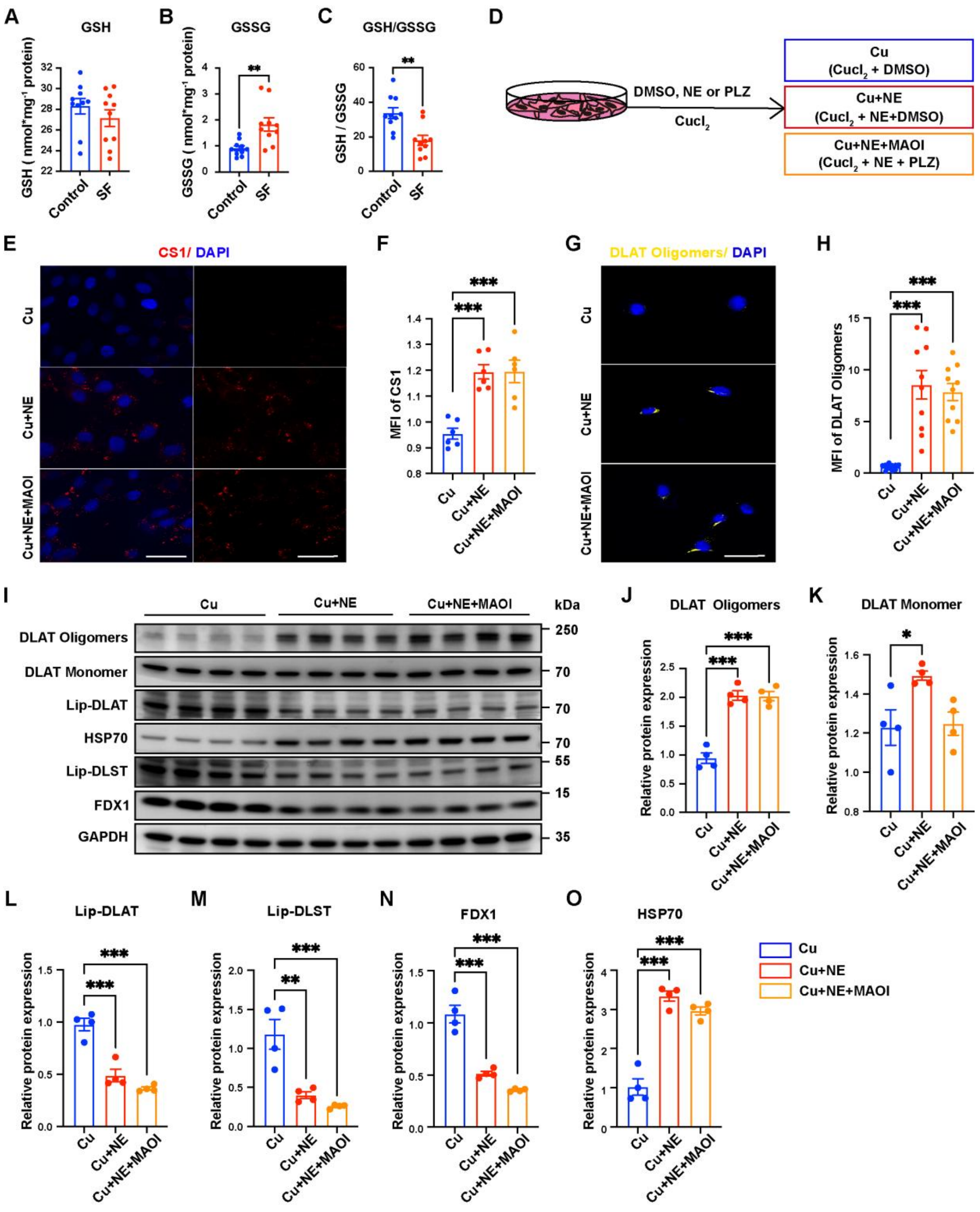

Fig. S4 Norepinephrine (NE) led to copper overload and cuproptosis in cardiomyocytes independent of monoamine oxidases inhibitor (MAOI). (A-C) The levels of GSH and GSSG and the GSH/GSSG ratio in mouse myocardial tissue were detected by ELISA (n=10 mice per group). **(D)** Schematic diagram of the experimental groups in the cell model. HL-1 cells were treated with 10 μM CuCl2 in the presence of solvent (DMSO), 1 μM NE

or 80 μM Phenelzine(PLZ) for 24 hours. **(E)** Representative images of copper overload immunofluorescence imaging by confocal microscopy. (red: Coppersensor1 (CS1; indicates intracellular copper ions), blue: DAPI). Scale bar=50 μm. **(F)** CS1 staining was quantified by calculating the mean fluorescence intensity (n=6 independent experiments). **(G)** Representative images of DLAT oligomer immunofluorescence imaging by confocal microscopy. Scale bar=50 μm. **(H)** DLAT oligomers levels were quantified by calculating the mean fluorescence intensity (n=10 cells examined over 5 independent experiments). **(I)** Validation of the levels of DLAT oligomers, lipoylated proteins, iron-sulfur cluster proteins and HSP70 by Western blotting after treating HL-1 cells with 10 μM $CuCl_2$ in the presence of NE (1 μM), DMSO and PLZ (80 μM) for 24 hours. **(J-O)** Statistical analysis of DLAT oligomers, DLAT monomers, Lip-DLST, FDX1 and HSP70. The data on these proteins were normalized to GAPDH (n=4 independent experiments). All data are presented as the mean ± s.e.m. The significance of differences was evaluated using one-way ANOVA. *$P<0.05$, **$P<0.01$, ***$P<0.001$.

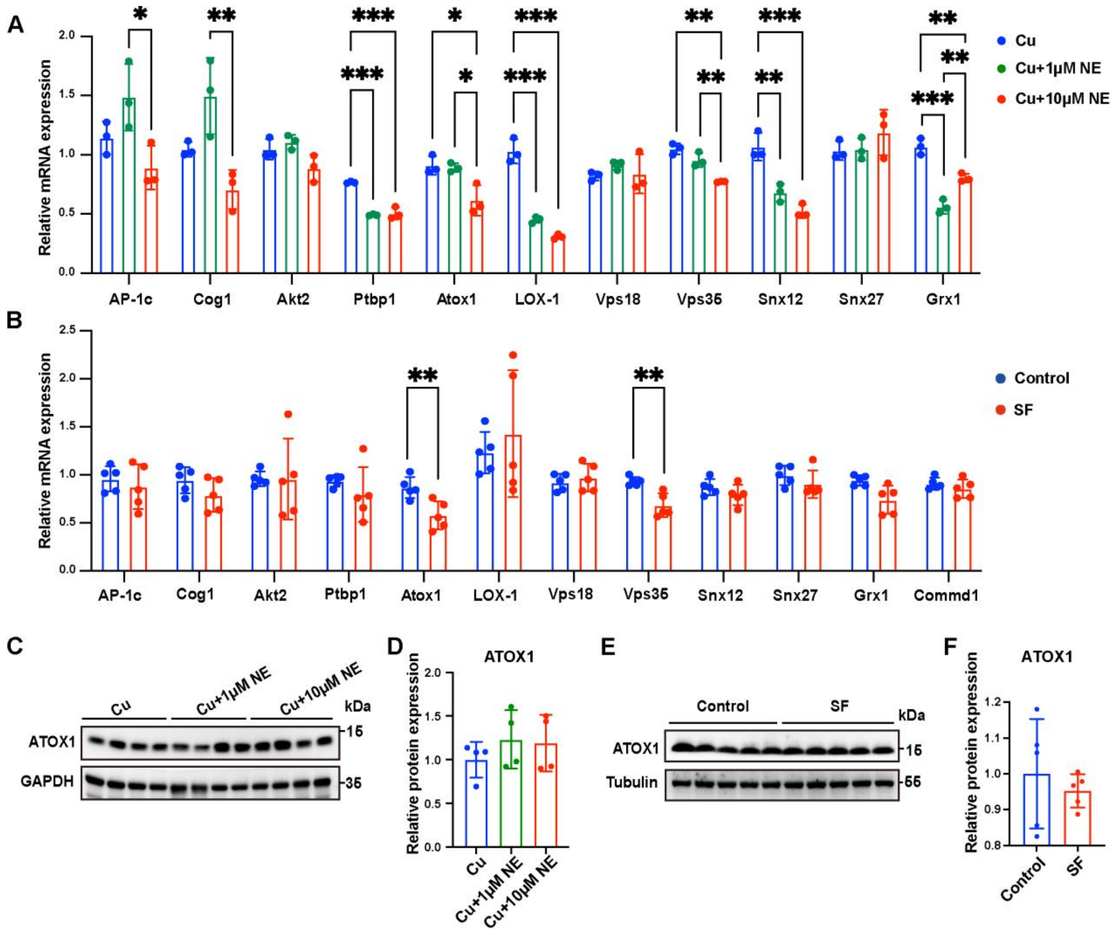

Fig. S5 Sympathetic signaling inhibited copper transport. (A) RT–qPCR was performed to analyze the mRNA levels of molecules affecting copper transport in HL-1 cells treated with 10 μM CuCl2 for 24 hours in the presence of DMSO, 1 μM or 10 μM NE (n=3 independent experiments). The significance of differences was evaluated using one-way ANOVA. **(B)** RT–qPCR analyses of the mRNA levels of molecules affecting copper transport in the myocardium of mice (n=5 mice per group). The significance of differences was evaluated using an unpaired t test. **(C)** Validation of the level of ATOX1 in HL-1 cells treated with 10 μM CuCl2 in the presence of DMSO or NE (1 μM, 10 μM) for 24 hours by Western blotting. **(D)** Statistical analysis of ATOX1 expression normalized to GAPDH (n=4 independent experiments).

The significance of differences was evaluated using one-way ANOVA. **(E)** Validation of the gene expression level of ATOX1 in the myocardium of mice (n=5). The significance of differences was evaluated using an unpaired t test. **(F)** Statistical analysis of ATOX1 expression normalized to GAPDH (n=5 mice per group). Data are presented as the mean ± s.e.m. *P<0.05, **P<0.01, ***P<0.001.

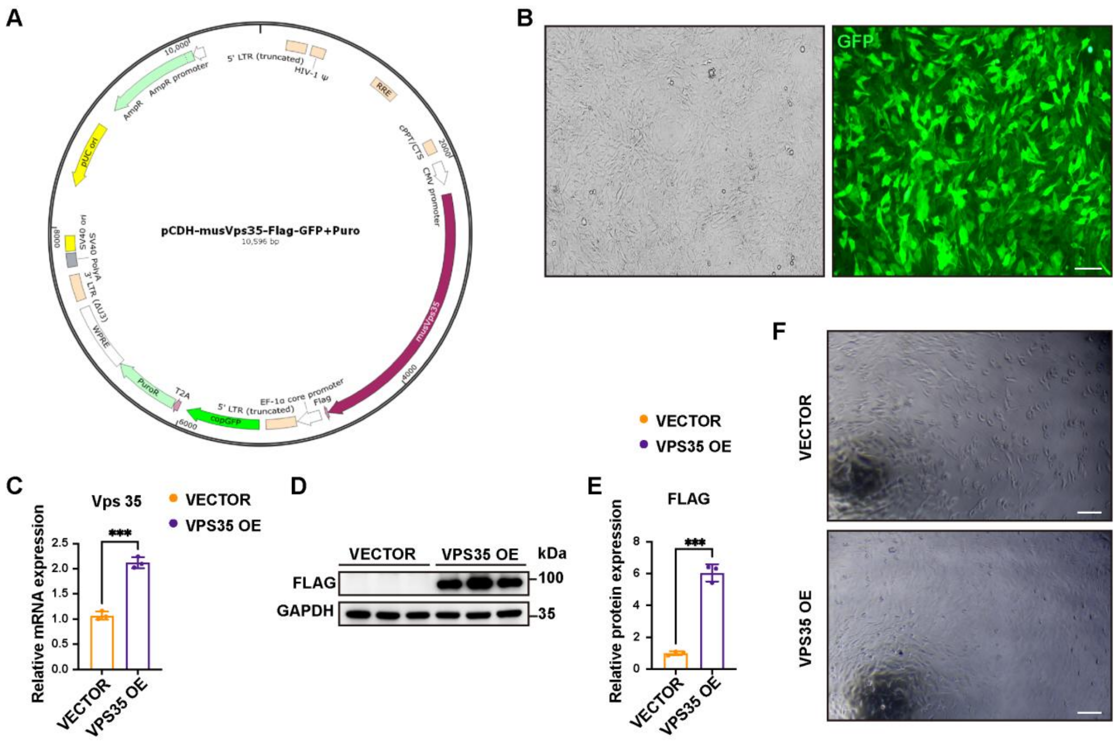

Fig. S6 A stable HL-1 cell line with VPS35 overexpression was constructed. (A) Schematic showing the construction of lentiviral vectors containing musVps35-Flag sequences (VPS35 OE). The corresponding control lentiviral vector (vector) construct was identical except for the absence of the musVps35-Flag sequence (picture not shown). **(B)** HL-1 cells that were successfully transfected with the VPS35 OE lentivirus were imaged under a

fluorescence microscope. Scale bar = 100 μm. **(C)** RT–qPCR analysis of Vps35 expression levels in HL-1 cells 96 h after transfection with lentivirus (n = 3 independent experiments). **(D)** Validation of the level of Flag in HL-1 cells 96 hours after transfection with lentivirus by Western blotting. **(E)** Statistical analysis of Flag expression normalized to GAPDH (n=3 independent experiments). **(F)** The morphology of vector and VPS35 OE hl-1 cells after 24 hours of coincubation with 10 μM NE and 10 μM $CuCl_2$ was observed by bright field microscopy. Scale bar = 100 μm. Data are presented as the mean ± s.e.m. The significance of differences was evaluated using an unpaired t test. ***P<0.001.

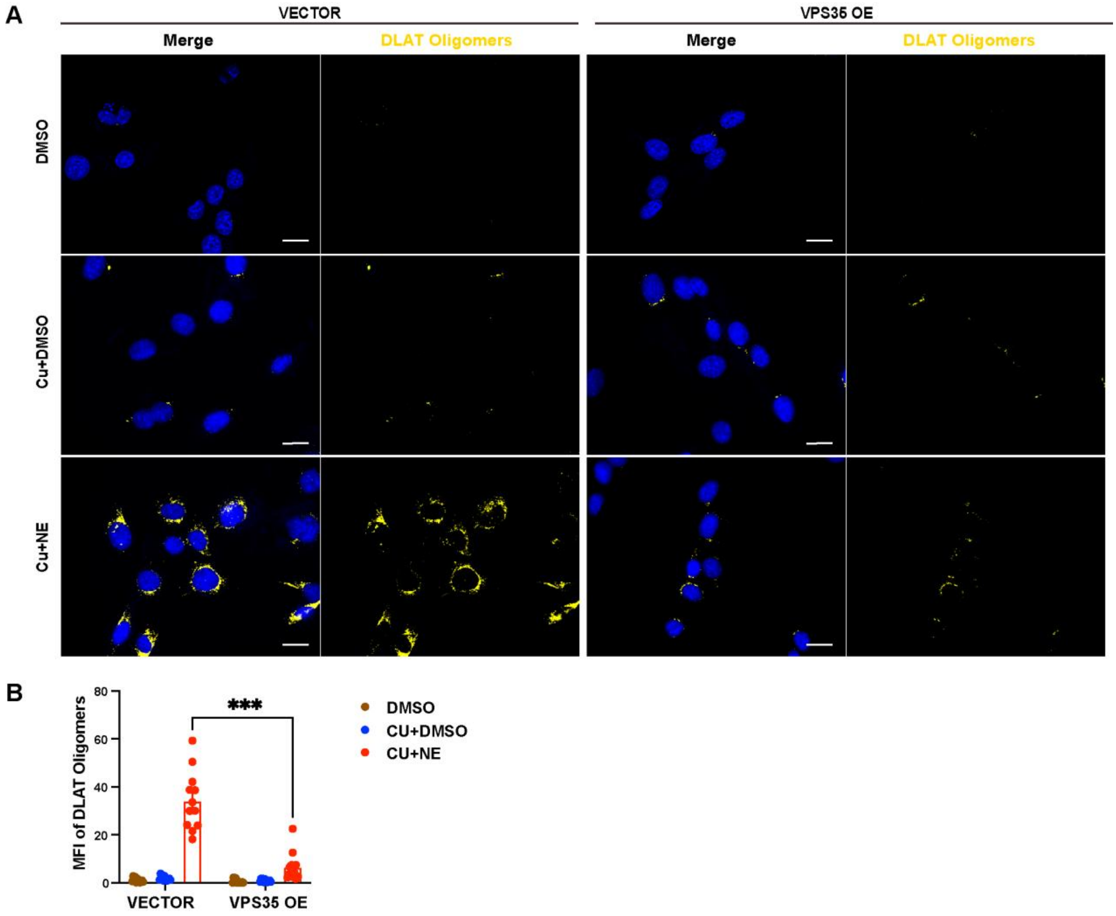

Fig. S7 Overexpression of VPS35 rescued cuproptosis caused by NE. (A) Representative images of DLAT oligomer immunofluorescence by confocal microscopy. Scale bar=20 μm. **(B)** The levels of DLAT oligomers were quantified by calculating the mean fluorescence intensity (n=12 cells examined over 4 independent experiments). All data are presented as the mean ± s.e.m. The significance of differences was evaluated using two-way ANOVA. *P<0.05, **P<0.01, ***P<0.001.

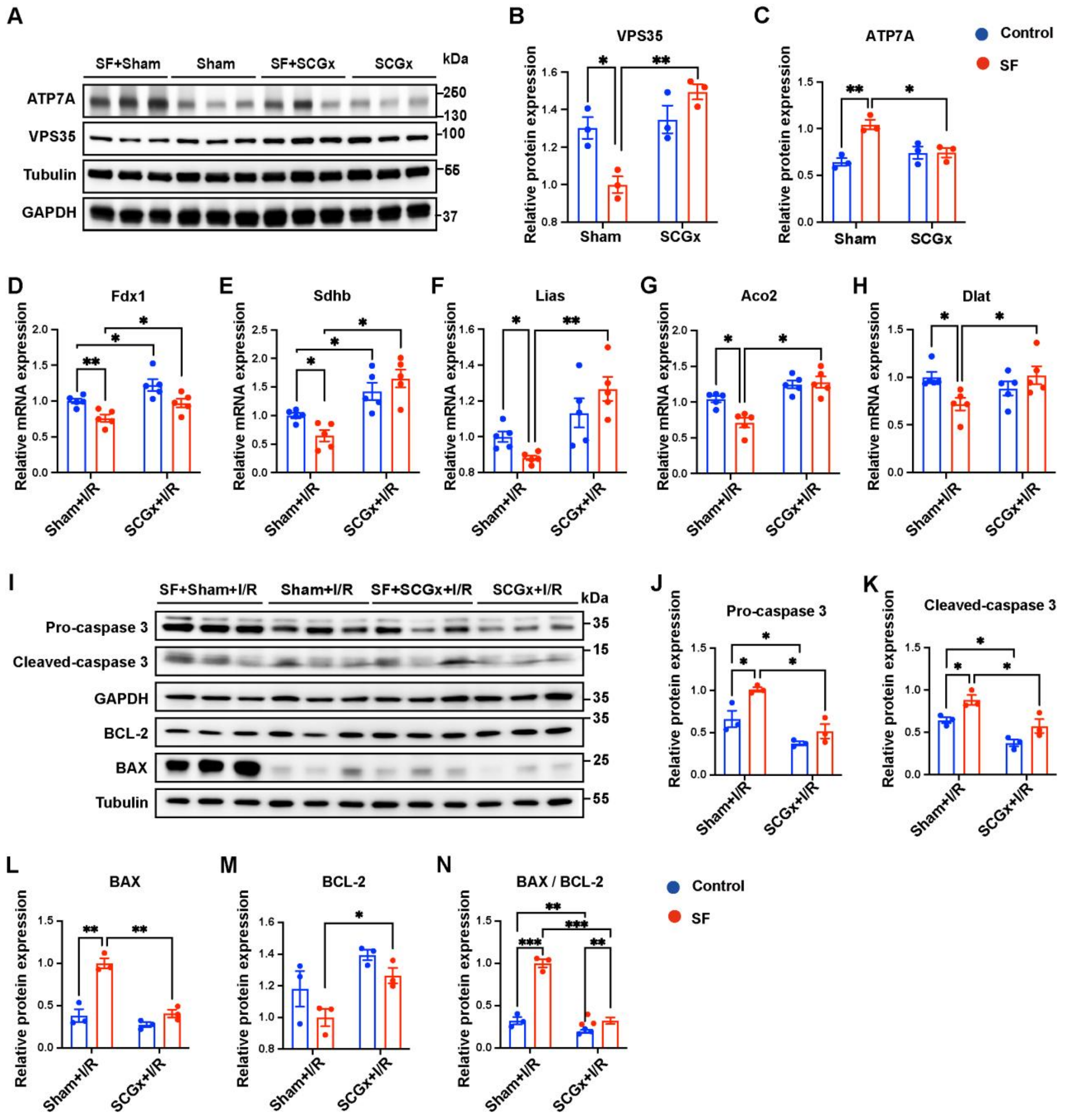

Fig. S8 SCGx attenuated copper homeostasis, cuproptosis and apoptosis in mice with SF. (A) Validation of the levels of ATP7A and VPS35 by Western blotting. **(B)** Statistical analysis of VPS35 expression, which was normalized to tubulin (n=3 mice per group). (**C)** Statistical analysis of ATP7A expression, which was normalized to GAPDH (n=3 mice per group). (**D-H)** RT–qPCR analysis of the mRNA levels of Fdx1, Sdhb, Lias, Aco2 and Dlat in the myocardium (n=5 mice per group). (**I)** Validation of the levels of pro-caspase 3, cleaved caspase 3, BAX and BCL-2 by Western blotting. **(J, K)** Statistical analysis of pro-caspase3 and cleaved caspase3 expression. Data on these proteins were normalized to GAPDH (n=3 mice per group). **(L, M)** Statistical analysis of BAX and BCL-2 expression. Data on these proteins were normalized to tubulin (n=3 mice per group). **(N)** Statistical analysis of the BAX/BCL-2 ratio (n=3 mice per group). Data are presented as the mean ± s.e.m. The significance of differences was evaluated using one-way ANOVA. *P<0.05, **P<0.01, *** p<0.001.

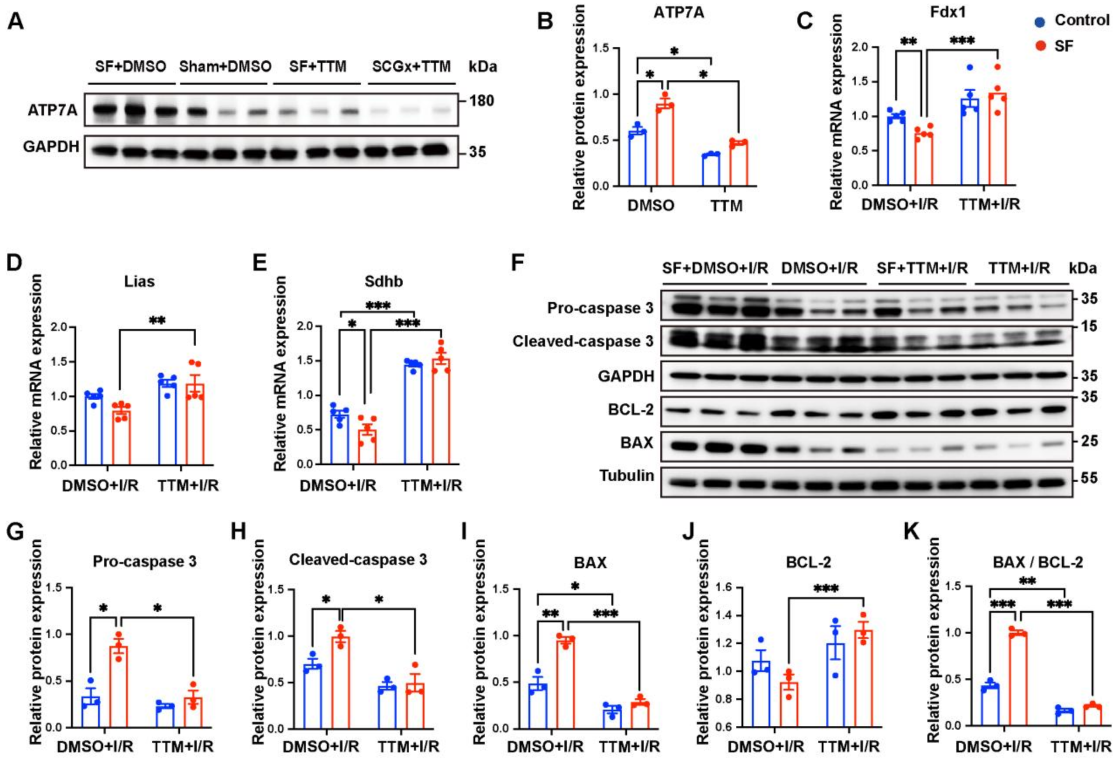

Fig. S9 TTM attenuated copper homeostasis, cuproptosis and apoptosis after MI/RI. **(A)** Validation of the level of ATP7A in the TTM- or DMSO-treated mouse myocardium by Western blotting. **(B)** Statistical analysis of ATP7A expression, which was normalized to GAPDH (n=3 mice per group). (**C-E)** RT–qPCR analyses of the mRNA levels of iron-sulfur cluster proteins in the myocardium (n=5 mice per group). **(F)** The protein expression of pro-caspase 3, cleaved caspase 3, BAX and BCL-2 in the myocardium was detected by Western blotting. **(G, H)** Statistical analysis of pro-caspase3 and cleaved caspase3 expression. Data on these proteins were normalized to GAPDH (n=3 mice per group). **(I, J)** Statistical analysis of BAX and BCL-2 expression. Data on these proteins were normalized to tubulin (n=3 mice per group). (**K)** Statistical analysis of the BAX/BCL-2 ratio (n=3 mice per group). Data are presented as the mean ± s.e.m. The significance of differences was evaluated

using one-way ANOVA. *P<0.05, **P<0.01, ***P<0.001.

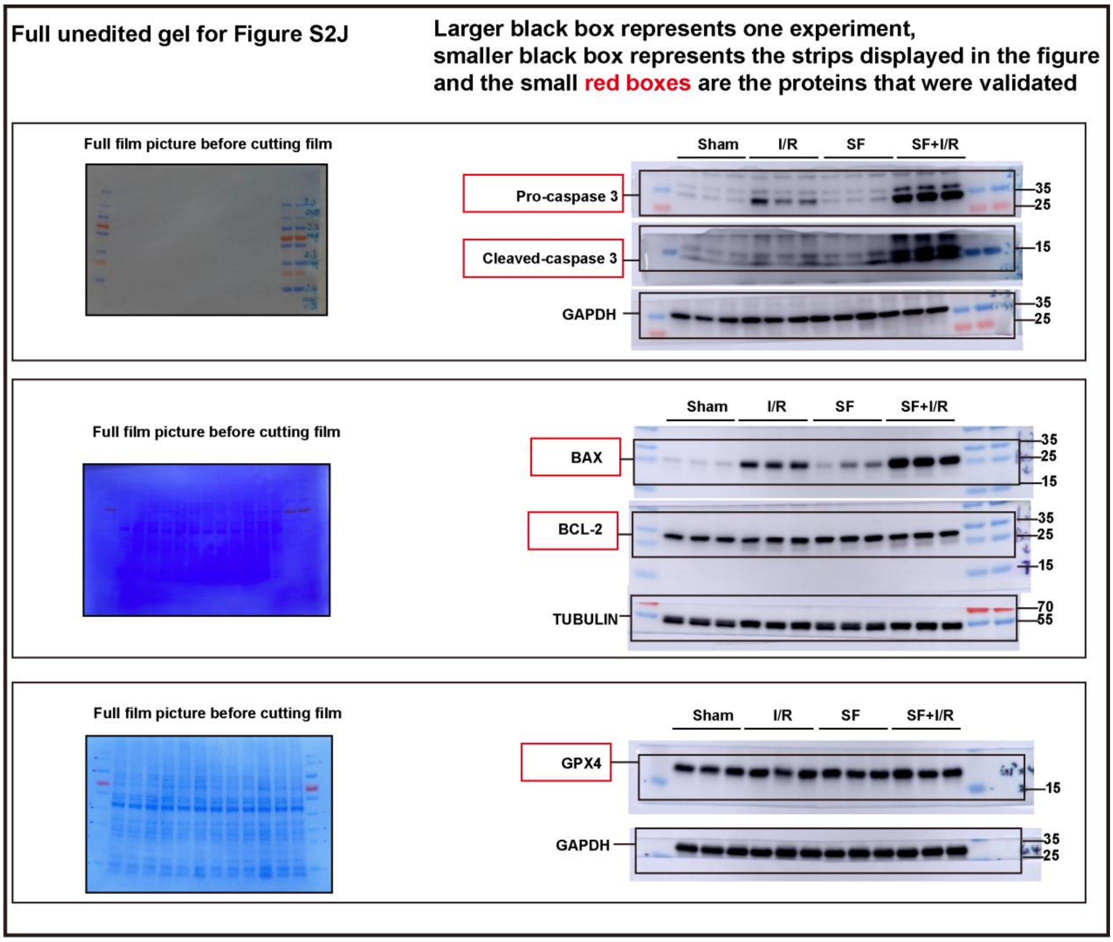

Full unedited gel for Figure S4I

Larger black box represents one experiment, smaller black box represents the strips displayed in the figure and the small red boxes are the proteins that were validated

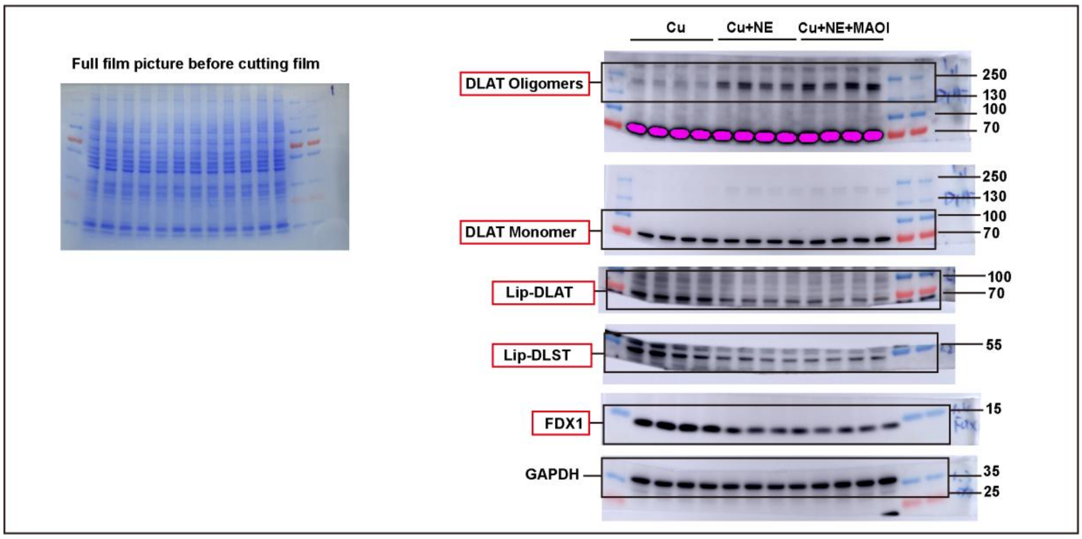

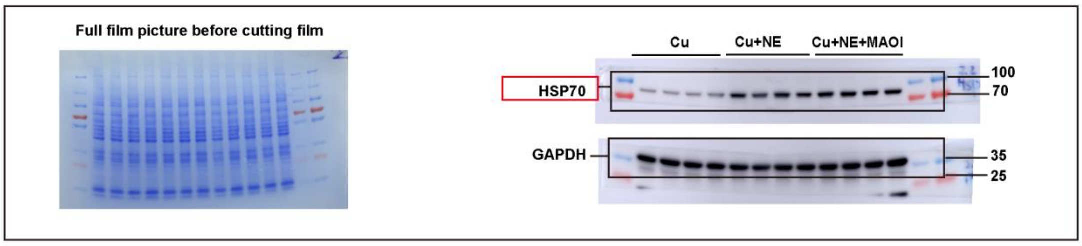

Full unedited gel for Figure S5C

Larger black box represents one experiment, smaller black box represents the strips displayed in the figure and the small red boxes are the proteins that were validated

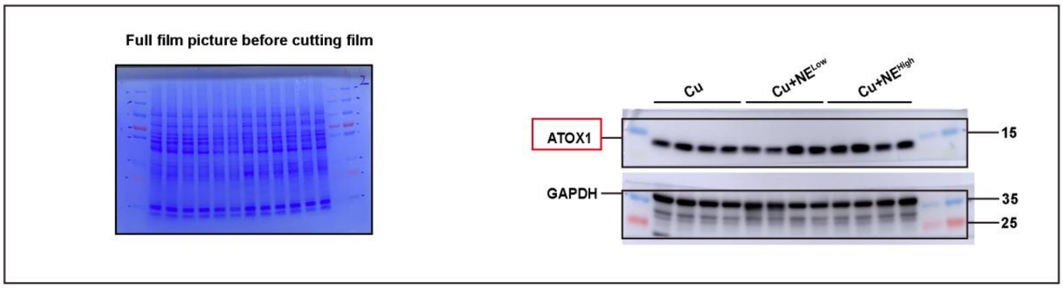

Full unedited gel for Figure S5E

Larger black box represents one experiment, smaller black box represents the strips displayed in the figure and the small red boxes are the proteins that were validated

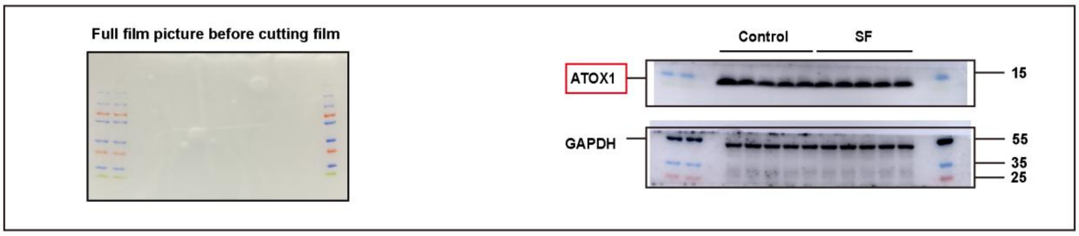

Full unedited gel for Figure S6D

Larger black box represents one experiment, smaller black box represents the strips displayed in the figure and the small red boxes are the proteins that were validated

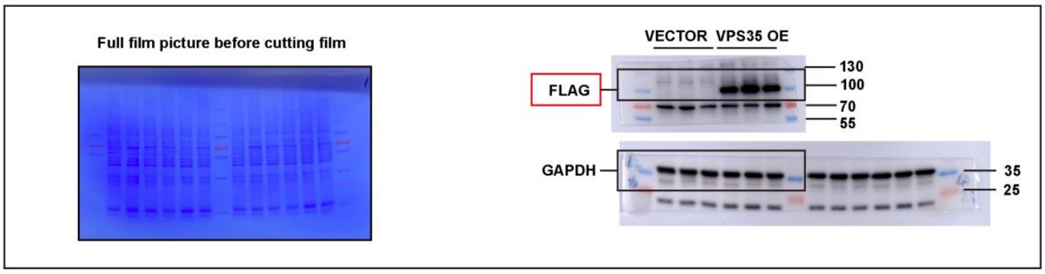

Full unedited gel for Figure S8A

Larger black box represents one experiment, smaller black box represents the strips displayed in the figure and the small red boxes are the proteins that were validated

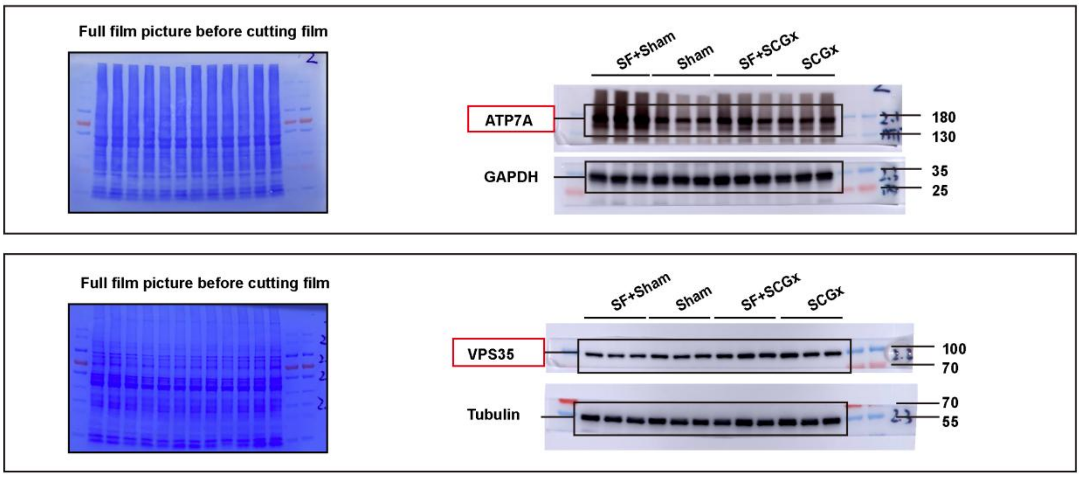

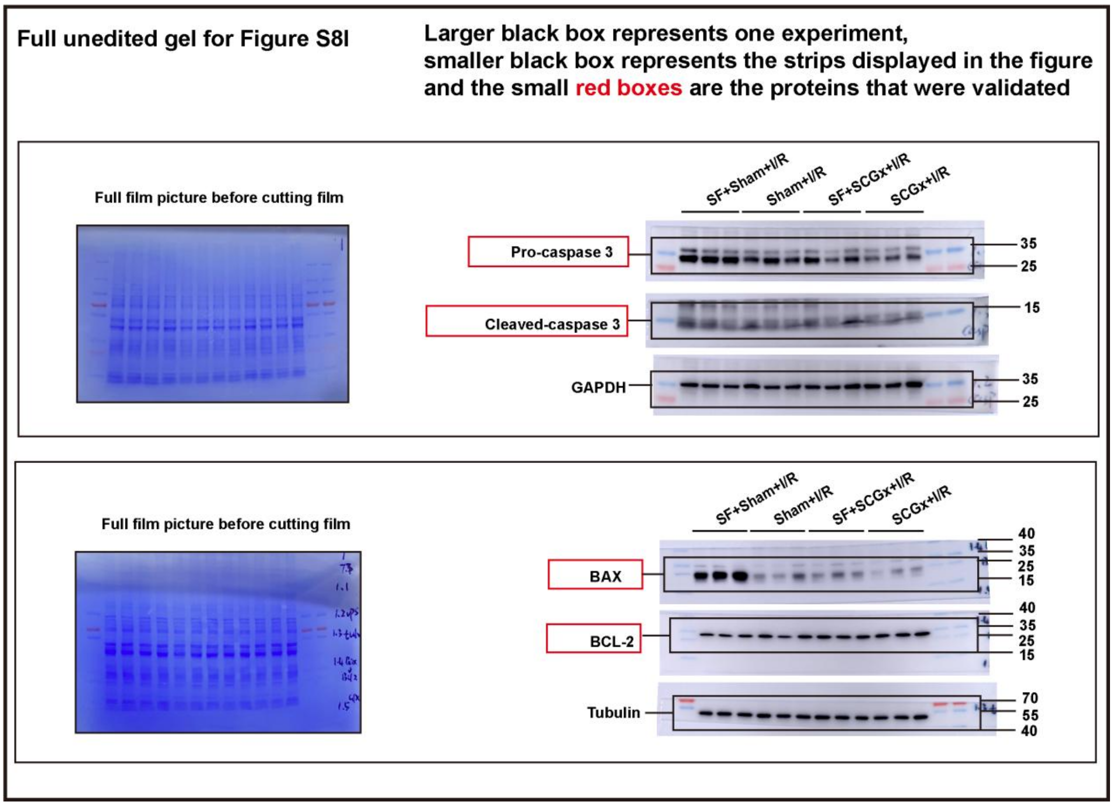

Full unedited gel for Figure S8I
Larger black box represents one experiment,
smaller black box represents the strips displayed in the figure
and the small red boxes are the proteins that were validated
Full film picture before cutting film
SF+Sham+I/R
Sham+I/R
SF+SCGx+I/R
SCGx+I/R
Pro-caspase 3
Cleaved-caspase 3
GAPDH
35
25
15
35
25
Full film picture before cutting film
SF+Sham+I/R
Sham+I/R
SF+SCGx+I/R
SCGx+I/R
BAX
BCL-2
Tubulin
40
35
25
15
40
35
25
15
70
55
40

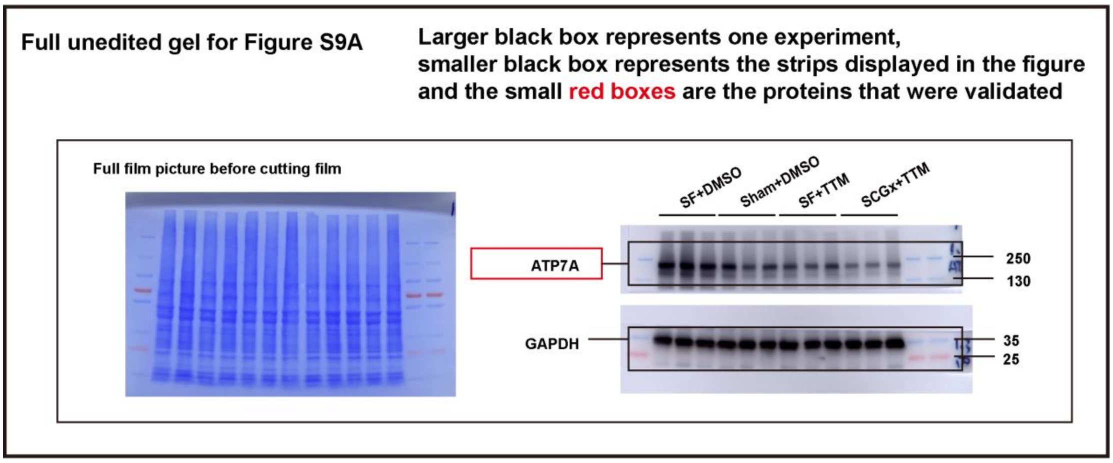

Full unedited gel for Figure S9A
Larger black box represents one experiment,
smaller black box represents the strips displayed in the figure
and the small red boxes are the proteins that were validated
Full film picture before cutting film
SF+DMSO
Sham+DMSO
SF+TTM
SCGx+TTM
ATP7A
GAPDH
250
130
35
25

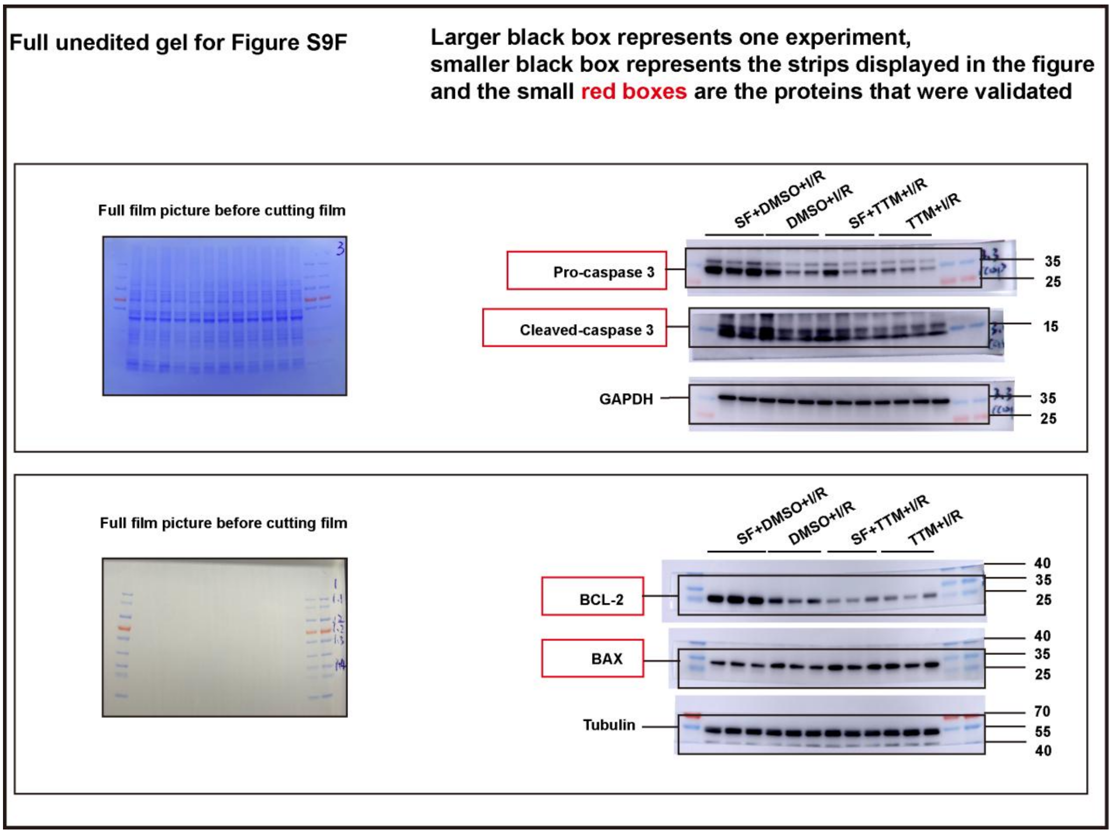
Full unedited gel for Figure S9F
Larger black box represents one experiment,
smaller black box represents the strips displayed in the figure
and the small red boxes are the proteins that were validated
Full film picture before cutting film
SF+DMSO+I/R
DMSO+I/R
SF+TTM+I/R
TTM+I/R
Pro-caspase 3
35
25
Cleaved-caspase 3
15
GAPDH
35
25
Full film picture before cutting film
SF+DMSO+I/R
DMSO+I/R
SF+TTM+I/R
TTM+I/R
BCL-2
40
35
25
BAX
40
35
25
Tubulin
70
55
40
