# Supplementary material for: Sleep fragmentation exacerbates myocardial ischemia‒reperfusion injury by promoting copper overload in cardiomyocytes
Source: Nat Commun. 2024 May 7;15:3834. doi: 10.1038/s41467-024-48227-y (PMC11076509; doi:10.1038/s41467-024-48227-y)
Supplement: Supplementary file 3 — Reporting Summary [file 41467_2024_48227_MOESM3_ESM.pdf]

nature portfolio

Corresponding author(s): DAPR NCOMMS-23-55586A

Last updated by author(s): Apr 10, 2024

Reporting Summary

Nature Portfolio wishes to improve the reproducibility of the work that we publish. This form provides structure for consistency and transparency in reporting. For further information on Nature Portfolio policies, see our Editorial Policies and the Editorial Policy Checklist.

Statistics

For all statistical analyses, confirm that the following items are present in the figure legend, table legend, main text, or Methods section.

n/a	Confirmed	
☐	☒	The exact sample size (*n*) for each experimental group/condition, given as a discrete number and unit of measurement
☐	☒	A statement on whether measurements were taken from distinct samples or whether the same sample was measured repeatedly
☐	☒	The statistical test(s) used AND whether they are one- or two-sided<br>*Only common tests should be described solely by name; describe more complex techniques in the Methods section.*
☒	☐	A description of all covariates tested
☐	☒	A description of any assumptions or corrections, such as tests of normality and adjustment for multiple comparisons
☐	☒	A full description of the statistical parameters including central tendency (e.g. means) or other basic estimates (e.g. regression coefficient) AND variation (e.g. standard deviation) or associated estimates of uncertainty (e.g. confidence intervals)
☒	☐	For null hypothesis testing, the test statistic (e.g. *F*, *t*, *r*) with confidence intervals, effect sizes, degrees of freedom and *P* value noted<br>*Give P values as exact values whenever suitable.*
☒	☐	For Bayesian analysis, information on the choice of priors and Markov chain Monte Carlo settings
☒	☐	For hierarchical and complex designs, identification of the appropriate level for tests and full reporting of outcomes
☒	☐	Estimates of effect sizes (e.g. Cohen's *d*, Pearson's *r*), indicating how they were calculated

Our web collection on statistics for biologists contains articles on many of the points above.

Software and code

Policy information about availability of computer code

Data collection	Confocal images were captured using Zeiss AirScan(LSM900) and Zeiss Apo Tome.2 with ZEN software (Carl Zeiss, v2009). Brightfield imaging was performed with a Nikon （N2Ti2-A） microscope and NIS-Elements Viewer software. Macroscopic fluorescence imaging of heart tissue was conducted using the AKOYA VECTRA POLARIS multispectral pathology imaging system. Cardiac electrophysiology data was collected using telemetry implants (ADInstruments, MT 10B) and LabChart software (8.1.24). Cardiac ultrasound data was acquired and measured using the portable ultrasound diagnostic device, M9 by Mindray. Metal ion detection was performed using Inductively Coupled Plasma Mass Spectrometry (ICP-MS) (Perkin Elmer NexION 2000). RT-qPCR data was collected using QuantStudio seven. Western blot bands were captured using the GE IMAGEQUANTMT 800 imaging system.
Data analysis	Softwares and scripts used are described in the Methods section and listed below as well.<br>Data analysis: The analysis of image data was performed using ImageJ（1.53a） and Fiji software （v2.9.0）. The ultrasound data obtained from the M9 by Mindray was analyzed using Microsoft (Version 16.8) and GraphPad Prism software (Version 9.2.0). The cardiac electrophysiology data was analyzed using the HRV module in LabChart （Version 8.1.24）.<br>Data representation: Figures were prepared with GraphPad Prism 9.2.0, Adobe InDesign 2021, and Adobe Illustrator 2021.

For manuscripts utilizing custom algorithms or software that are central to the research but not yet described in published literature, software must be made available to editors and reviewers. We strongly encourage code deposition in a community repository (e.g. GitHub). See the Nature Portfolio guidelines for submitting code & software for further information.

Data

Policy information about availability of data

All manuscripts must include a data availability statement. This statement should provide the following information, where applicable:

- Accession codes, unique identifiers, or web links for publicly available datasets
- A description of any restrictions on data availability
- For clinical datasets or third party data, please ensure that the statement adheres to our policy

The authors declare that the data supporting the findings of this study are available within the paper and the supplementary information files. Source data are provided with this paper. Any additional raw data will be available from the corresponding author upon reasonable request.

Research involving human participants, their data, or biological material

Policy information about studies with human participants or human data. See also policy information about sex, gender (identity/presentation), and sexual orientation and race, ethnicity and racism.

Reporting on sex and gender	NO human research participants in the study.
Reporting on race, ethnicity, or other socially relevant groupings	NO human research participants in the study.
Population characteristics	NO human research participants in the study.
Recruitment	NO human research participants in the study.
Ethics oversight	NO human research participants in the study.

Note that full information on the approval of the study protocol must also be provided in the manuscript.

Field-specific reporting

Please select the one below that is the best fit for your research. If you are not sure, read the appropriate sections before making your selection.

☒ Life sciences ☐ Behavioural & social sciences ☐ Ecological, evolutionary & environmental sciences

For a reference copy of the document with all sections, see nature.com/documents/nr-reporting-summary-flat.pdf

Life sciences study design

All studies must disclose on these points even when the disclosure is negative.

Sample size	No statistical method was used to predetermine the sample size. The sizes were determined to be as minimal as possible to reduce costs and ensure a statistically significant difference between experimental groups. Preliminary experiments were conducted to verify the reproducibility of statistical differences. For in vivo studies, each group contains at least three samples to ensure statistical validity.
Data exclusions	No data was excluded in this study.
Replication	Each experiment was done with multiple mice and each mouse was tested for multiple sessions. All attempts at replication of experiments were successful, as evident by the individual data points reported throughout the figures.
Randomization	Cells or mice were randomly assigned to different groups before treatments.
Blinding	The surgical operators, data collectors, and analysts were all blinded to the study groups.

Reporting for specific materials, systems and methods

We require information from authors about some types of materials, experimental systems and methods used in many studies. Here, indicate whether each material, system or method listed is relevant to your study. If you are not sure if a list item applies to your research, read the appropriate section before selecting a response.

Materials & experimental systems

n/a	Involved in the study
☐	☒ Antibodies
☐	☒ Eukaryotic cell lines
☒	☐ Palaeontology and archaeology
☐	☒ Animals and other organisms
☒	☐ Clinical data
☒	☐ Dual use research of concern
☒	☐ Plants

Methods

n/a	Involved in the study
☒	☐ ChIP-seq
☒	☐ Flow cytometry
☒	☐ MRI-based neuroimaging

Antibodies

Antibodies used

Information of all antibodies used were given in the Supplementary Table 2 and listed below as well.
Primary antibodies:
Rabbit Anti-Caspase-3 p12 (1:1000 dilution), ab179517 Abcam
Mouse Anti-PDC-E2 (1:400 dilution for WB, 1:200 dilution for IF), sc-271534 SANTA
Mouse anti-Hsp70 (1:1000 dilution), ab2787 Abcam
Rabbit anti-Lipoic Acid (1:2000 dilution), ab58724 Abcam
Rabbit anti-ADX (1:4000 dilution), ab108257 Abcam
Rabbit anti-GPX4 (1:4000 dilution), ab125066 Abcam
Mouse Anti-ATP7A (1:400 dilution for WB, 1:100 dilution for IF), sc-376467 SANTA
Rabbit anti-BAX (1:4000 dilution), ab32503 Abcam
Rabbit anti-BCL-2 (1:1000 dilution), ab196495 Abcam
Rabbit anti-VPS35 (1:10000 dilution), ab157220 Abcam
Rabbit anti-GAPDH (1:5000 dilution), YN5585 IMMUNOWAY
Rabbit anti-ATOX1 (1:5000 dilution), ab154179 Abcam
Rabbit anti-Flag (1:1000 dilution), K200001M Solarbio
Rabbit anti-β-Tubulin (1:5000 dilution), AP0064 Bioworld

Secondary antibodies:
Goat HRP-conjugated Affinipure Goat Anti-Rabbit IgG(H+L) Polyclonal antibody (Proteintech, SA00001-2, 1:10000 for Westen-blot);
Goat HRP-conjugated Affinipure Goat Anti-Mouse IgG(H+L) Polyclonal antibody (Proteintech, SA00001-1, 1:10000 for Westen-blot);
Goat Anti-Mouse IgG H&L (Alexa Fluor® 488), (abcam, ab150113);
Goat Anti-Rabbit IgG H&L (Alexa Fluor® 488), (abcam, ab150077);
Goat Anti-Mouse IgG H&L (Alexa Fluor® 594), (abcam, ab150116);

Validation

All antibodies used in this study are commercially available, verified and quality-tested by the suppliers.
Rabbit Anti-Caspase-3 p12 (1:1000 dilution), ab179517 Abcam （https://www.abcam.cn/products/primary-antibodies/caspase-3-p12-antibody-epr16888-ab179517.html）
Rabbit anti-GPX4 (1:4000 dilution), ab125066 Abcam(https://www.abcam.cn/products/primary-antibodies/glutathione-peroxidase-4-antibody-epncir144-ab125066.html);
Rabbit anti-BAX (1:4000 dilution), ab32503 Abcam(https://www.abcam.cn/products/primary-antibodies/bax-antibody-e63-ab32503.html);
Rabbit anti-VPS35 (1:10000 dilution), ab157220 Abcam(https://www.abcam.cn/products/primary-antibodies/vps35-antibody-epr11501b-ab157220.html);
Rabbit anti-ATOX1 (1:5000 dilution), ab154179 Abcam(https://www.abcam.cn/products/primary-antibodies/atox1-antibody-epr10352-ab154179.html);
Mouse Anti-PDC-E2 (1:400 dilution for WB, 1:200 dilution for IF), sc-271534 SANTA, (https://www.scbt.com/p/pdc-e2-antibody-b-2);
Mouse anti-Hsp70 (1:1000 dilution), ab2787 Abcam (https://www.abcam.cn/products/primary-antibodies/hsp70-antibody-5a5-ab2787.html);
Rabbit anti-Lipoic Acid (1:2000 dilution), ab58724 Abcam (https://www.abcam.cn/products/primary-antibodies/lipoic-acid-antibody-ab58724.html);
Rabbit anti-ADX (1:4000 dilution), ab108257 Abcam (https://www.abcam.cn/products/primary-antibodies/adx-antibody-epr4629-ab108257.html);
Mouse Anti-ATP7A (1:400 dilution for WB, 1:100 dilution for IF), sc-376467 SANTA (https://www.scbt.com/p/atp7a-antibody-d-9);
Rabbit anti-BCL-2 (1:1000 dilution), ab196495 Abcam (https://www.abcam.cn/products/primary-antibodies/bcl-2-antibody-ab196495.html);
Rabbit anti-GAPDH (1:5000 dilution), YN5585 IMMUNOWAY ();
Rabbit anti-Flag (1:1000 dilution), K200001M Solarbio ();
Rabbit anti-β-Tubulin (1:5000 dilution), AP0064 Bioworld ();
Goat HRP-conjugated Affinipure Goat Anti-Rabbit IgG(H+L) Polyclonal antibody (Proteintech, SA00001-2, 1:10000 for Westen-blot), (https://www.ptgcn.com/products/HRP-conjugated-Affinipure-Goat-Anti-Rabbit-IgG-H-L-secondary-antibody.htm);
Goat HRP-conjugated Affinipure Goat Anti-Mouse IgG(H+L) Polyclonal antibody (Proteintech, SA00001-1, 1:10000 for Westen-blot),

(https://www.ptgcn.com/products/HRP-conjugated-Affinipure-Goat-Anti-Mouse-IgG-H-L-secondary-antibody.htm);
Goat Anti-Mouse IgG H&L (Alexa Fluor® 488), (abcam, ab150113), (https://www.abcam.cn/products/secondary-antibodies/goat-mouse-igg-hl-alexa-fluor-488-ab150113.html);
Goat Anti-Rabbit IgG H&L (Alexa Fluor® 488), (abcam, ab150077), (https://www.abcam.cn/products/secondary-antibodies/goat-rabbit-igg-hl-alexa-fluor-488-ab150077.html);
Goat Anti-Mouse IgG H&L (Alexa Fluor® 594), (abcam, ab150116), (https://www.abcam.cn/products/secondary-antibodies/goat-mouse-igg-hl-alexa-fluor-594-ab150116.html?applications=78);

Eukaryotic cell lines

Policy information about cell lines and Sex and Gender in Research

Cell line source(s)	HL-1 cell line were purchased from Changsha Abiowell Biotechnology Co., Ltd
Authentication	Cell line was only authenticated by the morphology.
Mycoplasma contamination	Cell lines tested negative for mycoplasma.
Commonly misidentified lines (See ICLAC register)	None.

Animals and other research organisms

Policy information about studies involving animals; ARRIVE guidelines recommended for reporting animal research, and Sex and Gender in Research

Laboratory animals	Adult male C57BL/6 mice (6-8 weeks old, weighing 21-23g; male) were purchased from Hunan SJA Laboratory. All mice were raised in standard plastic cages under a temperature-controlled environment, with a 12-hour light/dark cycle, and had libitum access to food and water. All experimental procedures and protocols were approved by the Xiangya Hospital experimental animal Ethics Committee of Central South University (permit code: 2021111240) and adhered to the National Institute of Health Guide on the Care and Use of Laboratory Animals.
Wild animals	We didn't use wild animals in our study.
Reporting on sex	To avoid the effect of sex hormones secreted by female mice on the results, only male mice were included in the study.
Field-collected samples	This study did not involve samples collected from the field.
Ethics oversight	All experimental procedures and protocols were approved by the Xiangya Hospital experimental animal Ethics Committee of Central South University (permit code: 2021111240) and adhered to the National Institute of Health Guide on the Care and Use of Laboratory Animals.

Note that full information on the approval of the study protocol must also be provided in the manuscript.

Plants

Seed stocks	*Report on the source of all seed stocks or other plant material used. If applicable, state the seed stock centre and catalogue number. If plant specimens were collected from the field, describe the collection location, date and sampling procedures.*
Novel plant genotypes	*Describe the methods by which all novel plant genotypes were produced. This includes those generated by transgenic approaches, gene editing, chemical/radiation-based mutagenesis and hybridization. For transgenic lines, describe the transformation method, the number of independent lines analyzed and the generation upon which experiments were performed. For gene-edited lines, describe the editor used, the endogenous sequence targeted for editing, the targeting guide RNA sequence (if applicable) and how the editor was applied.*
Authentication	*Describe any authentication procedures for each seed stock used or novel genotype generated. Describe any experiments used to assess the effect of a mutation and, where applicable, how potential secondary effects (e.g. second site T-DNA insertions, mosiacism, off-target gene editing) were examined.*
